# A network-based predictive gene-expression signature for adjuvant chemotherapy benefit in stage II colorectal cancer

**DOI:** 10.1186/s12885-017-3821-4

**Published:** 2017-12-13

**Authors:** Bangrong Cao, Liping Luo, Lin Feng, Shiqi Ma, Tingqing Chen, Yuan Ren, Xiao Zha, Shujun Cheng, Kaitai Zhang, Changmin Chen

**Affiliations:** 1 Department of Basic Research, Sichuan Cancer Hospital & Institute, Sichuan Cancer Center, School of Medicine, University of Electronic Science and Technology of China, 55 Renmin Ave. Fourth Section, Chengdu, Sichuan 610041 China; 2 State Key Laboratory of Molecular Oncology, Department of Etiology and Carcinogenesis, Cancer Institute & Hospital, Peking Union Medical College and Chinese Academy of Medical Sciences, Beijing, China

**Keywords:** Colorectal cancer, Biomarkers, Adjuvant chemotherapy, 11-PPI-Mod

## Abstract

**Background:**

The clinical benefit of adjuvant chemotherapy for stage II colorectal cancer (CRC) is controversial. This study aimed to explore novel gene signature to predict outcome benefit of postoperative 5-Fu-based therapy in stage II CRC.

**Methods:**

Gene-expression profiles of stage II CRCs from two datasets with 5-Fu-based adjuvant chemotherapy (training dataset, *n* = 212; validation dataset, *n* = 85) were analyzed to identify the indicator. A systemic approach by integrating gene-expression and protein-protein interaction (PPI) network was implemented to develop the predictive signature. Kaplan-Meier curves and Cox proportional hazards model were used to determine the survival benefit of adjuvant chemotherapy. Experiments with shRNA knock-down were carried out to confirm the signature identified in this study.

**Results:**

In the training dataset, we identified 44 PPI sub-modules, by which we separate patients into two clusters (1 and 2) having different chemotherapeutic benefit. A predictor of 11 PPI sub-modules (11-PPI-Mod) was established to discriminate the two sub-groups, with an overall accuracy of 90.1%. This signature was independently validated in an external validation dataset. Kaplan-Meier curves showed an improved outcome for patients who received adjuvant chemotherapy in Cluster 1 sub-group, but even worse survival for those in Cluster 2 sub-group. Similar results were found in both the training and the validation dataset. Multivariate Cox regression revealed an interaction effect between 11-PPI-Mod signature and adjuvant therapy treatment in the training dataset (RFS, *p* = 0.007; OS, *p* = 0.006) and the validation dataset (RFS, *p* = 0.002). From the signature, we found that *PTGES* gene was up-regulated in CRC cells which were more resistant to 5-Fu. Knock-down of *PTGES* indicated a growth inhibition and up-regulation of apoptotic markers induced by 5-Fu in CRC cells.

**Conclusions:**

Only a small proportion of stage II CRC patients could benefit from adjuvant therapy. The 11-PPI-Mod as a potential predictor could be helpful to distinguish this sub-group with favorable outcome.

**Electronic supplementary material:**

The online version of this article (10.1186/s12885-017-3821-4) contains supplementary material, which is available to authorized users.

## Background

Colorectal cancer (CRC) is one of the most common malignancies, and is among the leading causes of cancer-related death worldwide. The incidence and mortality of CRC have been rising during the past two decades in China. It was estimated that the newly diagnosis of CRC is 376,300 and approximately 191,000 people died in China in 2015 [1]. Surgery is the foundation of curative treatment for localized CRC, but approximately 25% of patients with AJCC stage II (or Dukes’ B) and nearly 45% of those with Stage III suffered recurrence after surgical resection [2]. Postoperative adjuvant chemotherapy was helpful to improve relapse free survival (RFS) of stage III patients [3, 4]. However, the benefit from adjuvant chemotherapy in Stage II CRC patients without lymph node metastasis is controversial. Routine clinical and pathological characteristics failed to predict RFS in many Stage II patients who received adjuvant chemotherapy [5]. The proper decision of whether a patient with Stage II disease should receive adjuvant chemotherapy would be important for improving prognosis.

Recent years, a series of molecular or genetic markers were identified as significant prognostic factors for CRC, including Microsatellite instability (MSI), Loss of heterozygosity (LOH), 18q deletion, *KRAS* mutations, and *BRAF* mutations et al. [6, 7]. However, the usefulness of these markers in predicting survival benefit of adjuvant chemotherapy is unclear. The defective DNA mismatch repair (dMMR) feature was correlated with good prognosis, and the patients with dMMR could not benefit from 5-Fu based adjuvant chemotherapy in stage II-III CRCs [8, 9]. In the proficient mismatch repair (pMMR) sub-group, the survival benefit of adjuvant chemotherapy was only observed in patients with stage III disease, but not in stage II sub-groups [9]. A multicentre randomized trail QUASAR was assigned to explore the survival benefit from adjuvant chemotherapy for patients with CRC at low risk of recurrence [10]. The QUASAR trial demonstrated that the 5-Fu based chemotherapy could improve survival of patients with stage II CRC. However, the 5-year absolute improvement of survival for adjuvant chemotherapy was only 3.6% [10]. Hutchins et al. analyzed the MMR status in the QUASAR trial, and found that the MMR status provided only prognostic value but not predictive significance for adjuvant chemotherapy in stage II CRCs [11]. Thus, for patients with stage II CRC of pMMR, novel predictive biomarkers are required for predicting outcome benefit of adjuvant chemotherapy.

Gene-expression profiles were widely used in prognostic signature development for CRC [2, 12–15]. Whereas, minimal concordance in overlapping of gene lists identified in these studies was observed. The human protein-protein interaction (PPI) network is a complex biological network composed of a lot of known or unknown pathways, and has been proposed to be informative in the identification of cancer biomarkers when being integrated with gene-expression profiles [16–19]. Compared with gene signature, function related PPI network might provide higher predictive accuracy and more reproducibility between different cohorts [17]. In addition, sub-modules (sub-networks) derived from PPI network can identify the tightly shared common biological themes, which will provide insight into new therapeutic strategies.

In this study, the gene-expression profiles of stage II CRCs of pMMR were analyzed by integration of PPI network from the Human Protein Reference Database (HPRD) [20]. A set of effective PPI sub-modules was identified for predicting the outcome benefit of 5-Fu based adjuvant chemotherapy. This signature was further validated in an independent dataset, and confirmed with CRC cell lines experimentally.

## Methods

### Patients and characteristics

A total of 297 patients with stage II (or Duke’s stage B) colorectal cancer were analyzed in this study. The training dataset (*n* = 212) was collected from the Gene Expression Omnibus (GEO) dataset GSE39582 [15], with the following criteria: a) American Joint Committee on Cancer (AJCC) stage II; b) tumors were characterized as pMMR; c) with follow-up information. There were 127 males and 85 females, and with a median age of 69 years old (range from 25 to 94 years old). Of these, 50 patients received Fluorouracil (5-Fu) based adjuvant chemotherapy after surgery resection, 162 patients received surgical treatment alone. The median followed-up time of this dataset is 4.7/5.3 years from the surgery date for RFS and overall survival (OS) respectively. The six molecular subtypes of CRCs identified by Marisa et al. was involved in the training dataset [15].

The validation dataset was a subset of the GEO dataset GSE14333 [21], including 85 patients with Duke’s Stage B colorectal cancer and follow-up information. The median age of these patients was 70 years old, with a range from 30 to 92 years old. There were 45 males and 40 females in this dataset, 13 patients received standard 5-Fu based adjuvant chemotherapy, and 72 ones received surgical treatment alone. The median RFS time of this dataset is 3.3 years from surgery date.

### Modularity analysis of protein-protein interaction network

PPI network was downloaded from the HPRD (Release 9) [22]. The whole PPI network was processed and analyzed using the R package of “igraph”. In details, replicated connections between two proteins were reduced to one unique interaction, the loops (connections between a protein and itself) were removed. The adjacency matrix of the network was used to calculate the general topological overlap matrix (GTOM) with 2-step common neighbors as previously described [23]. Unsupervised hierarchical clustering analysis was carried out using the 1-GTOM as distance matrix and complete linkage. Clusters (sub-modules) of the hierarchical dendrogram were detected by R package “dynamicTreeCut” [24], with parameters of max tree height of 0.6, minimum module size of 5 proteins, and deep split method.

### Gene expression data processing and GSVA profile transformation

Gene expression data (“cel” files of Affymetrix Human Genome U133 Plus 2.0 microarrays) of the selected samples were downloaded from GEO database. The gene expression profiles were normalized using the “RMA” method. “PMA” callings were detected by R package “affy” for the training and validation dataset respectively. Probes that were characterized as “Present” in more than 20% tumor samples were retained, resulting in 28,810 and 26,324 probes for the training and validation dataset respectively. Probe annotation was performed by the “hgu133plus2.db” package from Bioconductor, resulting in 13,274 unique Entrez gene ids for the training dataset, and 12,721 genes for the validation dataset. The 12,209 genes overlapped between the training dataset and validation dataset were employed in the subsequent analysis. A flowchart about data processing was shown in Additional file 1: Figure S1.

The PPI sub-modules were mapped onto the gene expression files based on Entrez gene ids. The Gene Set Variation Analysis (GSVA) [25] was employed to detection the variation value of the PPI sub-modules in each dataset, using the R package “GSVA” [25].

### Feature selection, predictive modeling, and independent validation for adjuvant chemotherapy related sub-groups

Cox’s proportional hazards model was used to test the interaction effect between adjuvant chemotherapy status and the PPI sub-modules on RFS of patients. The Benjamini and Hochberg’s [26] FDR < 0.05 for the interaction effect (chemotherapy & PPI sub-module group) was considered significant. The significant sub-modules were used to identify sub-groups of samples by unsupervised hierarchical clustering, with the distance of 1-Pearson’s correlation coefficient, and the complete linkage.

The sub-modules with the most importance and optimal predictive performance for the identified sub-groups were defined by the Random Forest feature selection algorithm using R package “varSelRF” [27], with the following parameters: 5000 trees in the first forest, 3000 trees in the iterative forests, and excluding 20% of variables at each iteration. The final solution was selected with the smallest number of PPI sub-modules whose “Out-of-Bag” (OOB) error rate is within standard error of the minimum error rate of all iterative forests [27]. For the PPI-sub-module predictor, the trained class probability was utilized for receiver operating characteristic (ROC) curve analysis. The areas under the ROC curves (AUC) with 95% confidence interval (CI) were calculated by the R package “pROC” [28]. Finally, the optimal PPI sub-module prediction model was validated in the validation dataset.

### Network visualization and biological annotation of selected PPI sub-modules

The R package “igraph” was used for network visualization. The biological and functional annotations of the 11 sub-modules were analyzed by the online tool DAVID [29, 30], using the Gene Ontology (GO) and the KEGG database. The Benjamini’s adjusted *p*-value <0.05 was considered as significant.

### Cell culture and treatment

All CRC cells were purchased from the American Type Culture Collection (ATCC) and the cell bank of Chinese Academy of Sciences. Cells were cultured in DMEM/F12 and RPMI-1640 medium with 10% Fetal Bovine Serum (FBS), and incubated at 37 °C with 5% CO_2_. Cell viability assay: cells were seeded in 96-well plates and treated with 5-Fu at 500 μM, 100 μM, 20 μM, 4 μM, 800 nM, 160 nM, 32 nM and 0 (6 wells/treatment) for 72 h. Cell viability was detected with Cell Counting Kit-8 (Dojindo, Kumamoto, Japan; Cat #CK04), the absorbance at 450 nm was recorded by iMark microplate reader (Bio-Rad, CA, USA). Lentivirus production and viral transduction were described as previously [31]: lentivirus was packaged by transfecting 3 μg lentiviral vector mixed with 2.7 μg helper plasmid pCMV-dR8.91 and 0.3 μg envelope plasmid (VSV-G) to 293 T cells by X­tremeGene HP (Roche, Basel, Switzerland; Cat # 6366236001). Cells with about 35% confluent were infected with virus and 10 μg/ml polybrene. After 24 h, cells were selected with fresh media containing puromycin at 2 μg/ml for 48 h. Cells were harvested and divided into two parts: a) treated with DMSO at a final concentration of 0.1% and 5-Fu at 5 μM for 16 h, and then harvested for Western blotting; b) for cell proliferation assay at 0, 24, 48, 72 h respectively. Lentiviral based short hairpin RNA (shRNA) constructs were purchased from Genechem Co. Ltd. (Shanghai, China): PTGES-shRNA-1, clone ID 44673, target sequence GGGCTTCGTCTACTCCTTT; PTGES-shRNA-2, clone ID 44674, target sequence ACGACATGGAGACCATCTA; Scramble, clone ID CON077, target sequence TTCTCCGAACGTGTCACGT.

### RNA extraction and RT-qPCR

Total RNA from CRC cell lines was isolated using RNeasy mini Kit (Qiagen, Duesseldorf, Germany; Cat # 74104), and then quantified by NanoDrop 2000 (Thermo, MA, USA). 100 ng of total RNA was subjected to RT-qPCR analysis with the iTaq Universal SYBR One-Step Kit (Bio-Rad, CA, USA; Cat #1725150) on the CFX-Connect Real-Time PCR Detection System (Biorad, CA, USA) following the manufacturer’s instructions. The primers are as follows: *PTGES*, forward sequence, ACCCTTTTGTCGCCTGGAT, reverse sequence, GTAGGTCACGGAGCGGATG; *GAPDH* (endogenous control), forward sequence, ACCCAGAAGACTGTGGATGG, reverse sequence, TTCAGCTCAGGGATGACCTT. Average cycle threshold (Ct) of the triplicate experiments for each sample was used for the subsequent analysis. The gene expression was calculated using the 2^-ΔΔCt^ method [32], where ΔCt = Ct_target gene_– Ct_endogenous_, and ΔΔCt = ΔCt_individual sample_ – ΔCt_reference sample_.

### Western blotting

Cells were lysed with cell lysis buffer (CST, MA, USA; Cat #9803) in the presence of protease inhibitors. 40 μg of total protein were electrophoresed on 12% SDS-PAGE and electrophoretically transferred onto a PVDF membrane, blocked with 5% skim milk at room temperature (RT) for one hour. Membranes were later probed with different primary antibodies overnight at 4 °C. The membranes were washed for 5 min three times in TBS with 0.1% Tween-20 and then incubated with horseradish peroxidase-conjugated mouse (Cat #1706516, Biorad, CA, USA) or rabbit (Cat #1706515, Biorad, CA, USA) secondary antibodies at RT for one hour. The membranes were washed three times for 5 min in TBS with 0.1% Tween-20, and then visualized with the Lumi-Light Western Blotting Substrate (Roche, Basel, Switzerland; Cat #12015200001) on the 5200 chemiluminescence imager (Tanon, Shanghai, China). The following primary antibodies were purchased: mouse anti-PTGES (Santa Cruz, CA, USA; Cat #sc-166,309), rabbit anti-Cleaved Caspase-3 (CST, MA, USA; Cat #9661), mouse anti-Cleaved PARP-1 (Santa Cruz, CA, USA; Cat #sc-56,196), mouse anti-GAPDH (ZSGB-BIO, Beijing, China; Cat #TA-08).

### Other statistical methods

The associations between the 11-PPI-Mod sub-groups and other clinical variables (age, gender, tumor location, et al.) were estimated by Pearson’s Chi-squared test with Yates’ continuity correction. The univariate analysis for different clinical variables, or multivariate analysis for assessing interaction effect between adjuvant chemotherapy and other clinical parameters were performed using Cox’s proportional hazards model. The Kaplan-Meier curve and the log-rank test were employed to compare the RFS and OS of patients in different groups. The significance of RT-qPCR data was calculated by unpaired Student’s *t*-test. All of these statistical methods were two-sides, and performed by R software.

## Results

**Fig. 1:**
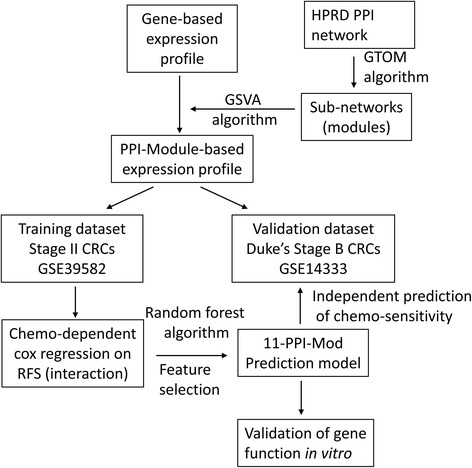
An overview of the workflow in this study. In details, a set of promising protein-protein interaction sub-modules were identified from the HPRD database. The gene-based expression profile was transformed into PPI-module-based profiles by the GSVA algorithm. Using Cox regression model and Random forest algorithm, we established a prediction model named 11-PPI-Mod in the training dataset. This signature was also validated in another independent validation dataset. In addition, in vitro experiments were performed to validate one of the predictive genes in terms of chemoresistance

A workflow for thist study is depicted in Fig. 1.

### Identification of protein-protein interaction sub-modules by GTOM

**Fig. 2:**
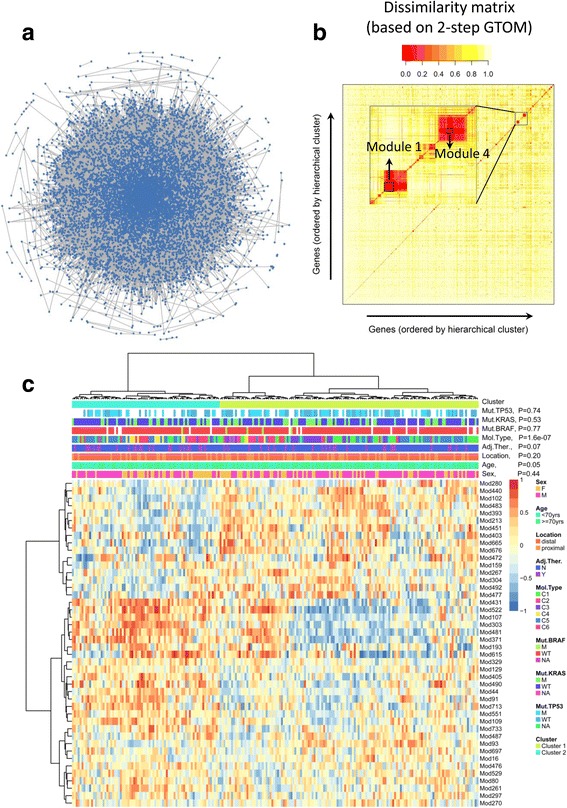
Identification of protein-protein interaction (PPI) sub-modules in stage II CRC patients. **a** The whole PPI network from HPRD database (Release 9) is visualized. Each point represents a protein, and lines for interactions between proteins. **b** The sub-modules derived from GTOM matrix. The dissimilarity matrix (1-GTOM2) is shown. Proteins (or genes) are ordered by unsupervised hierarchical clustering analysis based on the dissimilarity matrix. Two sub-modules (Module 1 and 4) are magnified. **c** The GSVA profiles (bottom heat map) of 44 PPI sub-modules in the training dataset. Unsupervised hierarchical clustering analysis is performed for PPI sub-modules (rows, hierarchical tree on the left panel) and patients (columns, hierarchical tree on the upper panel) respectively. The middle panel indicates annotations (clinical or genetic variables) of the patients, with *p* values estimated by Chi-square test for correlations between these variables and the two clusters. Abbreviations for clinical or genetic variables: F, female; M, male; yrs., years; Adj.Ther., adjuvant chemotherapy; N, no; Y, yes; Mol.Type, the six molecular subtypes defined by the original article; Mut.BRAF, Mut.KRAR and Mut.TP53, somatic mutation status of BRAF/KRAS/TP53; M, mutation; WT, wild type; NA, not available

A protein-protein interaction network composed of 9501 proteins (nodes) and 36,963 interactions (edges) was extracted from the HPRD (Fig. 2a). To identify the PPI sub-modules with tightly co-regulated proteins, a general topological overlap matrix (GTOM) of the 9501 proteins was calculated based on 2-step common neighbors in the PPI network. Unsupervised hierarchical clustering and followed branch detection analysis revealed that 5580 out of 9501 proteins were divided into 740 distinct sub-modules (Fig. 2b). The remained 3921 proteins did not reach the criteria (GTOM dissimilarity <0.6, or sub-module protein number ≥ 5) of the unsupervised hierarchical clustering analysis, and were excluded in the subsequent analysis. Among the identified 740 sub-modules, the median of protein/gene number was 7, with a range from 5 to 48.

Of the 740 PPI sub-modules, 734 had been mapped onto the gene expression profiles of the training and validation datasets. Gene Set Variation Analysis (GSVA) was employed to transform the single-gene-based gene expression profile to PPI-sub-module-based GSVA profile, with 734 rows (sub-modules) and 212/85 columns (sample numbers in training/validation dataset). The GSVA profiles were generated, and applied in the subsequent modeling and prediction analysis.

### Stratification of CRC sub-groups by expression profiles of PPI sub-modules

In the training dataset of stage II CRC of pMMR (*n* = 212), among the diverse clinical or genetic variables, only pathological T stage showed prognostic values for RFS (Additional file 1: Table S1, *P* = 0.019). For OS, age (*P* < 0.01) and the “C3” molecular subtype (*P* = 0.019) achieved statistical significance by Cox analysis (Additional file 1: Table S2). Meanwhile, the patients who received adjuvant chemotherapy showed no benefit based on either RFS (Additional file 1: Table S1, *P* = 0.28) or OS (Additional file 1: Table S2, *P* = 0.64), compared to those with surgical treatment alone. We also tested the interaction effect between adjuvant chemotherapy and other clinical variables, but none of these showed a significant result for RFS (Additional file 1: Table S1). Only KRAS status achieved statistical significance for OS (Additional file 1: Table S2, *P* = 0.016).

In order to identify sub-group of patients who may benefit from adjuvant chemotherapy, the PPI sub-modules were preselected by Cox model in analyzing the interaction effect between treatment and the GSVA profiles. As a result, 44 sub-modules were associated with different RFS benefit from adjuvant chemotherapy (interaction effect, *FDR* < 0.05). With an unsupervised clustering program, patients were clustered into two major sub-groups (Fig. 2c), with 135 patients in Cluster 1 and 77 patients in Cluster 2. The two clusters were correlated with six molecular subtypes (Chi-square test, *P* < 0.001) and patients age (Chi-square test, *P* = 0.05), but not correlated with gender, tumor location, BRAF /KRAS / TP53 mutation status or adjuvant chemotherapy treatment (Chi-square test, *P* > 0.05) (Fig. 2c).

### Construction of predictor for the sub-groups identified in stage II CRC

**Fig. 3:**
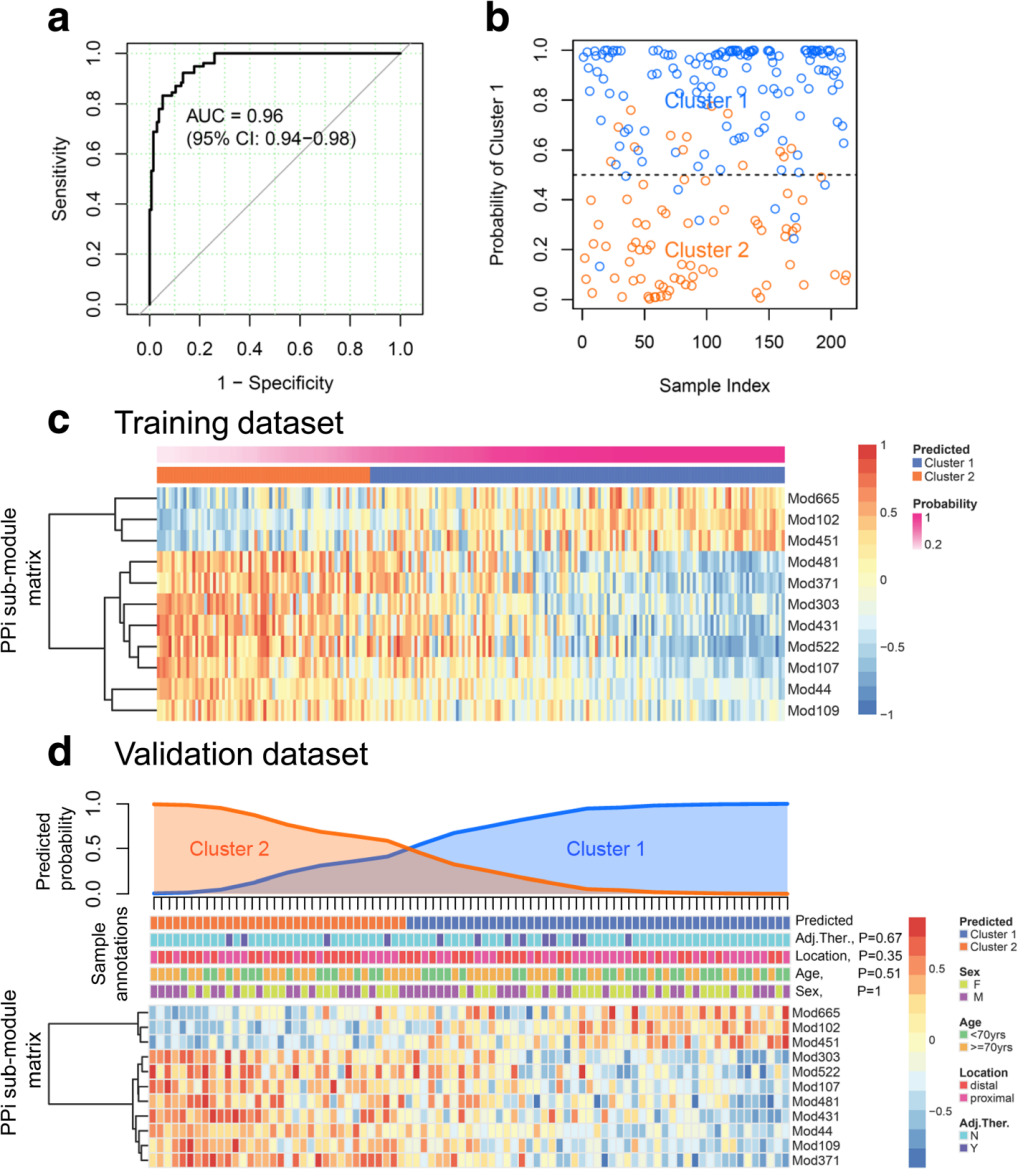
Predictor construction for sub-group classification of patients. **a** Receiver operating characteristic curve (ROC) of the 11-PPI-Mod predictor in the training dataset. The predicted probability of Cluster 1 is adopted for ROC analysis. The area under the curve (AUC) and its 95% confidence interval (CI) are shown. **b** Distribution of individuals in the training dataset. The x-axis represents the patient index. Y-axis is the predicted probability of Cluster 1. Each point indicates an individual patient, with different colors labeling for Cluster 1 (blue) and Cluster 2 (red). The dotted line indicates the cut-off (probability = 0.5) of the predictor. **c** The GSVA profiles (heat map) of the optimized 11 PPI sub-modules in the training dataset. PPI sub-modules are organized by unsupervised hierarchical tree on the left side. Patients are sorted by the predicted probability of Cluster 1 (the upper panel). **d** Sub-group classification of patients in the validation dataset. The upper panel indicates the predicted probability of Cluster 1 (blue) or Cluster 2 (red) of patients. The middle panel indicates annotations of the patients, with *p* values estimated by Chi-square test for correlations between each clinical variable and predicted two clusters. The heat map in the bottom panel shows the GSVA profiles of 11-PPI-Mod. PPI sub-modules are organized by unsupervised hierarchical tree on the left side. Patients are sorted by the predicted probability of Cluster 1. Abbreviations: F, female; M, male; yrs., years; Adj.Ther., adjuvant chemotherapy; N, no; Y, yes

The random forest algorithm revealed that the 44 PPI sub-modules exhibited a significant importance for predicting the two sub-groups, compared with the simulated results (Additional file 1: Figure S2A). Among the iterative random forests, the minimum OOB error rate is 0.085 ± 0.019 (mean ± sd). Within this error rate range, the combination of 11 PPI sub-modules (OOB error rate = 0.099 ± 0.021) reached the criteria of the smallest feature number and was finally selected for constructing the prediction model (Additional file 1: Figure S2B). This model was referred to as a 11-PPI-Mod predictor, of which the area under the predictive ROC curve was 0.96 (95% CI: 0.94–0.98) (Fig. 3a). Using predicted probability >0.5 as the cut-off, 140 patients were predicted as Cluster 1, and 72 patients as Cluster 2, with an overall accuracy of 90.1% (191/212, Fig. 3b). In the 11-PPI-Mod predictor, three sub-modules were up-regulated in Cluster 1, and eight sub-modules were up-regulated in Cluster 2 (Fig. 3c).

The 11-PPI-Mod predictor constructed in the training dataset was further applied on the validation dataset (*n* = 85). Of the 85 patients, 51 of them were classified as Cluster 1, and the rest of 34 patients were grouped into Cluster 2 (Fig. 3d). The predicted sub-groups were not associated with age, gender, tumor location, or adjuvant chemotherapy group (Chi-square test, *P* > 0.1) (Fig. 3d).

### Outcome benefit of adjuvant chemotherapy stratified by 11-PPI-mod predictor

**Fig. 4:**
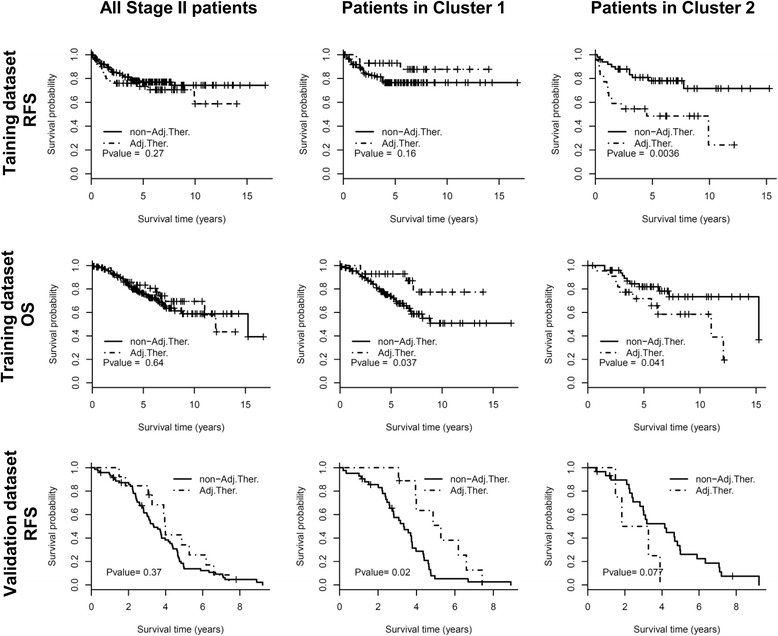
Survival analysis of adjuvant chemotherapy in stage II CRC patients. The upper and middle panels show RFS and OS in the training dataset respectively. The bottom panel shows RFS in the validation dataset. The entire cohort of stage II patients (the left column), Cluster 1 sub-group (the middle column) and Cluster 2 sub-group (the right column) stratified by 11-PPI-Mod predictor are analyzed respectively. The RFS/OS of patients who received adjuvant chemotherapy are compared to those of patients without adjuvant therapy. The *p* value is calculated by log-rank test. Abbreviations: Adj.Ther., with adjuvant chemotherapy; non-Adj.Ther., without adjuvant chemotherapy

In the training dataset, the survival benefits from adjuvant chemotherapy were diverse in different sub-groups predicted by 11-PPI-Mod. There was no difference on RFS between patients with or without adjuvant chemotherapy when considering all stage II CRCs (log-rank test, *P* = 0.27). A trend toward RFS benefit was observed in the Cluster 1 sub-group (*P* = 0.16). Patients in Cluster 2 who received adjuvant chemotherapy showed even worse RFS than those without it (*P* = 0.004) (Fig. 4, the upper panel). For OS, those who received adjuvant chemotherapy showed no distinct prognosis considering the entire cohort (*P* = 0.64). Patients who received adjuvant chemotherapy in Cluster 1 demonstrated better outcome (*P* = 0.037), but patients who received adjuvant chemotherapy in Cluster 2 showed a worse outcome (*P* = 0.041) (Fig. 4, the middle panel). Multivariate Cox regression revealed a significant interaction effect between 11-PPI-Mod sub-groups and adjuvant chemotherapy treatment based on both RFS (Additional file 1: Table S1, *P* = 0.007) and OS (Additional file 1: Table S2, *P* = 0.006).

In the validation dataset, univariate Cox analysis indicated that none of the clinical variables or the 11-PPI-Mod predictor could predict RFS (*P* > 0.05) (Additional file 1: Table S3). However, the 11-PPI-Mod sub-groups showed a predictive value for RFS benefit of adjuvant therapy. There was no significant difference on RFS between patients with or without adjuvant chemotherapy within entire cohort (log-rank test, *P* = 0.37). However, the adjuvant chemotherapy treatment was associated with improved RFS in Cluster 1 sub-group (*P* = 0.02), but a trend of decreased RFS in Cluster 2 sub-group (*P* = 0.07), compared with the surgery treatment alone (Fig. 4, the bottom panel). Multivariate Cox model indicated a significant interaction effect between adjuvant chemotherapy treatment and the sub-groups predicted by 11-PPI-Mod (Additional file 1: Table S3, *P* = 0.002).

### The biological significance of the 11-PPI-mod predictor

**Fig. 5:**
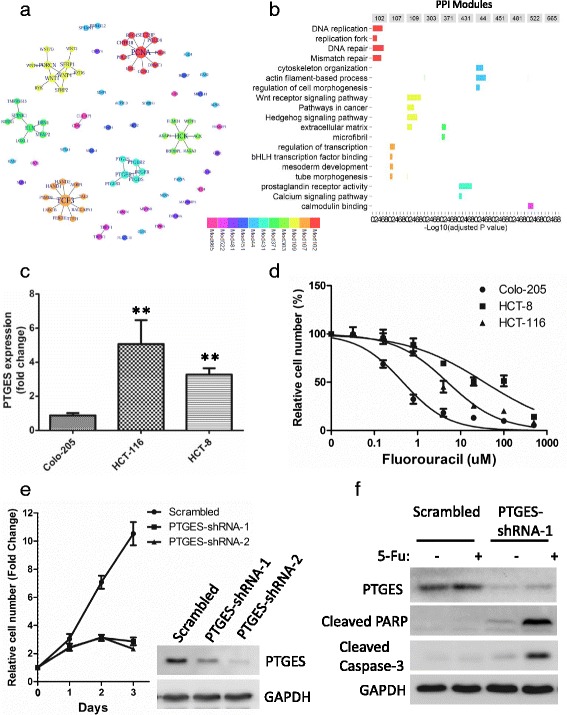
Biological significance of genes in the 11-PPI-Mod predictor. **a** Network visualization of the 11-PPI-Mod predictor. Each node is a single gene/protein, and each line indicates an interaction between two genes/proteins. The size of nodes represents interaction degree, and the color indicates different sub-modules (see legends). **b** Gene set enrichment analysis of each PPI sub-module of 11-PPI-Mod predictor. The –Log_10_transformed adjusted *p* values (x-axis) of significant GO terms or KEGG pathways (y-axis) are shown, across different PPI sub-modules (columns). **c** Gene expression of *PTGES* in three CRC cell lines by qRT-PCR. Relative expression (fold change) of *PTGES* was calculated with *GAPDH* as reference gene, and normalized to that of Colo-205 cells. Data represents means and standard deviations (SD, error bars) from three independent experiments, with triplicate amplifications for each experiment. The *P* value was calculated by unpaired Student’s t-test (two sided). ** HCT-116 or HCT-8 vs Colo-205, *p* < 0.05. **d** Dose-response of Fluorouracil (5-Fu) on the growth effect of three CRC cell lines. Cells were treated with DMSO or different concentration of 5-Fu for 72 h. The viable cell number was determined by MTT assay. Data is plotted as mean +/− SD of 3 independent experiments with sextuplets for each experiment. **e** Growth curves of HCT-116 cells. Relative cell number (y-axis) is normalized to 0 h. Mean with standard deviation (SD, error bars) for each time point is shown. Data represents results from three independent experiments with triplicates. Western-blotting indicates the Knocked down expression of *PTGES*. **f** Effect of *PTGES* on apoptosis of HCT-116 cells. Cells infected with lentiviruses encoding *PTGES*-targeting or scrambled shRNAs were treated with 5-Fu (5 μM) or DMSO for 16 h. Cell lysates were subjected to Western-blotting analysis for two apoptosis markers

There were 86 genes in the 11 selected PPI sub-modules (Additional file 1: Table S4). 50 genes from six sub-modules were directly connected into six sub-networks according to protein-protein interactions (Fig. 5a). In other five sub-modules, most of the proteins were not connected directly (Fig. 5a), the high modularity of these proteins probably results from the tight co-regulation with their common neighbors. Moreover, gene set enrichment analysis showed that the 11 sub-modules were related to diverse GO terms and KEGG pathways (Fig. 5b). For instance, Mod102 was significantly correlated with DNA replication and DNA repair. Mod44 was enriched in cytoskeleton organization and regulation of cell morphogenesis. Mod107 was referred to bHLH transcription factor binding and embryonic development. Mod109 was mostly related with Wnt signaling pathway and Hedgehog signaling pathway, and Mod431 was enriched in prostaglandin receptor activity.

### PTGES from 11-PPI-mod is associated with chemoresistance in CRC cells

Among the 86 genes in the 11-PPI-Mod predictor, *PTGES* gene was further investigated in CRC cells. The mRNA levels of *PTGES* were significantly higher in HCT-116 (Fold Change = 4.99, *P* = 0.04) and HCT-8 (Fold Change = 3.71, *P* = 0.01) than that of Colo-205 cells (Fig. 5c). Meanwhile, Colo-205 (IC_50_ = 0.46 μM, 95% CI: 0.33–0.63) was more sensitive to chemotherapeutic agent Fluorouracil (5-Fu) than HCT-116 cells (IC_50_ = 4.94 μM, 95% CI: 3.4–7.18) and HCT-8 cells (IC_50_ = 35.39 μM, 95% CI: 21.37–58.61) (Fig. 5d). Knock-down expression of *PTGES* by shRNA resulted in significant growth inhibition of HCT-116 cells (Fig. 5e). Furthermore, compared to the scrambled control, knock-down of *PTGES* showed dominant elevation in apoptosis markers of cleaved Caspase-3 and PARP induced by 5-Fu (Fig. 5f).

## Discussion

Nearly 25–30% of patients with stage II (or Dukes’ B) CRC would relapse after surgical resection [2]. However, the clinical benefit of post-surgical adjuvant chemotherapy for Stage II CRC is controversial. It was reported that the absolute risk reduction for recurrence of adjuvant chemotherapy with 5-fluorouracil (5-FU) in stage II patients is only 3–5% in 5 years [5], resulting in a great challenge in determining whether a patient with stage II CRC should receive adjuvant chemotherapy. It is necessary to explore novel predictive signatures to identify patients who most likely benefit from adjuvant chemotherapy. In the present study, we developed a predictive model named 11-PPI-Mod by integrating the HPRD PPI network and the gene expression profiles of stage II CRCs. Patients classified as Cluster 1 sub-group might get a better outcome after adjuvant chemotherapy. In contrast, in Cluster 2, patient with adjuvant chemotherapy would receive no benefit, or even worse outcome. In the training dataset, although the improvement of RFS by chemotherapy did not achieve statistical significance in Cluster 1 subgroup, a significantly reduced outcome was observed in the Cluster 2 subgroup (Fig. 4, upper panel). Similarly, a reversed trend of outcome between Cluster 1 and Cluster 2 was found in the validation dataset (Fig. 4, bottom panel). The reversed outcome trend indicated a potential interaction effect between 11-PPI-Mod subgroups and treatment, which was also confirmed by the Cox analysis (Additional file 1: Table S1–3). Furthermore, we showed that a gene identified by the 11-PPI-Mod is correlated with chemoresistance in CRC cells.

There are several genetic or clinical risk factors for stage II CRC, including MMR status (or MSI), T4 stage, poor tumor differentiation, intestinal obstruction, detected lymph node <10 [5]. The dMMR tumors indicate a lack of efficacy of 5-Fu based adjuvant therapy, while the outcome benefit of chemotherapy is unclear in stage II pMMR tumors [9]. However, other risk factors showed no predictive value for adjuvant chemotherapy. In this study, we focus on the pMMR tumors in the training dataset to develop the predictive signature for adjuvant chemotherapy. Because of the lack of MMR status in the validation dataset, we validated the signature using the whole dataset of stage II patients. Generally, only a small proportion of all CRC tumors are characterized as dMMR [9, 33], which may have little effect on the predictive value of our signature. The results from the training and validation datasets suggested that our signature were effective in patient stratification for chemotherapy regardless of MMR status.

Hutchins et al. reported that Kras mutation was a prognostic marker for poor RFS, but could not predict benefit from chemotherapy in stage II CRC [11]. We found that Kras gene mutation carried out a significant interaction effect with chemotherapy treatment based on OS. Patients with wild type Kras were more likely to benefit from chemotherapy, compared with those harboring Kras mutation (Additional file 1: Figure S3). The chemo-derived benefit in patients with wild type Kras was restricted in Cluster 1 but not in Cluster 2, based on stratification of patients by our 11-PPI-Mod signature. Meanwhile, the benefit of chemotherapy was not significant anymore in Cluster 1 with Kras mutation. Thus, a combination of Kras status and 11-PPI-Mod signature would be more precise in predicting the benefit of adjuvant chemotherapy in stage II CRC (Additional file 1: Figure S3).

Previous studies have reported several gene-expression signatures associated with the prognosis of stage II CRC patients [2, 5, 12–14]. These studies usually identified prognostic genes to group patients into high/low risk subgroups, and the individuals at high-risk group were therefore proposed to receive more benefit from chemotherapy [2, 13, 14]. One limitation of this strategy is that most of the identified genes would reflect the prognostic significance, but little is related to drug response/resistance. Our approach focused on the interaction effect between gene variables and therapeutic status on the patient outcome. This method would identify both the prognostic genes and the drug sensitivity/resistance molecules. Despite the fact that the 11-PPI-Mod predictor showed no prognostic significance within all patients, Cluster 1 is associated with poor outcome in patients without chemotherapy (Additional file 1: Figure S4). In the chemotherapy arm, Cluster 2 inversely showed worse RFS and OS than Cluster 1 (Additional file 1: Figure S4), suggesting that the genes up-regulated in Cluster 2 might relate to drug-resistance.

Many of the biological processes or pathways identified in the present study correlate with chemotherapeutic sensitivity in cancer cells. DNA replication and cellular proliferation (annotated by Mod102) related protein Ki-67 and cyclin D1 could predict benefit from adjuvant chemotherapy in colon cancer [34, 35]. Mod109 represented Wnt/beta-catenin pathway, which was involved in resistance to chemotherapy in osteosarcoma [36] and hepatocellular carcinoma [37]. Meanwhile, the *TCF3* sub-module (Mod107) was enriched in transcriptional regulators of epidermal and embryonic stem cells [38], consistent with that stem-like properties were associated with decreased benefit from chemotherapy in colorectal cancer [39, 40]. These data collectively suggest that a variety of genes in the identified sub-modules may reflect the chemoresistance of cancer cells, and might be novel therapeutic targets for improving patient outcome.

In this study, a PPI module (Mod431) of prostaglandin (PG) receptor activity was significantly correlated with limited chemotherapy benefit for stage II colorectal cancer. Prostaglandins (PGs) are essential mediators of the inflammatory process and may play critical roles in proliferation, apoptosis, invasiveness, angiogenesis and inflammatory response during carcinogenesis [41–44]. Prostaglandin E synthase family has three members (*PTGES*, *PTGES2*, *PTGES3*), which catalyze the oxidoreduction of prostaglandin endoperoxide H2 (PGH2) to prostaglandin E2 (PGE2) [45]. Over expression of the three family members were found in glioma [45]. The protein levels of *PTGES* and *PTGES2* were correlated with poor prognosis in CRC patients [46]. Intratumoral hypoxia microenvironment could induce *PTGES* expression through a HIF-1alpha-dependent manner [47]. It has been reported that hypoxia contributes to chemoresistance in various cancers, including CRC [48–50]. However, whether or not *PTGES* plays a role in chemoresistance has been unknown yet. We presumed that the dysregulation of *PTGES* (or PG receptor pathway) would serve as a novel mechanism for chemoresistance in CRC. Depending on our result, the activation of PG pathways is predictive for chemoresistance in stage II CRC patients. Our experimental results demonstrated that knock-down of *PTGES* resulted in proliferation inhibition and enhanced apoptosis in response to 5-Fu in CRC cells. These data suggest that the PG pathway and related key molecules would serve as potential predictive biomarkers for adjuvant chemotherapy, and *PTGES* might be a novel target for sensitizing CRC to chemo-agents.

PPI network has been reported to be informative in developing cancer biomarkers when being integrated with gene-expression profiles [16–19]. A network-based approach may identified much more robust signatures, compared with the gene-based methodology [17]. The HPRD PPI network is constructed by a lot of experimentally validated protein interactions, which reflect known or unknown biological pathways [22]. Using a network-base approach, we identified a set of significant PPI sub-modules that were correlated with survival benefit of adjuvant chemotherapy in stage II CRC. These sub-modules might be related to either well-known or novel biological mechanisms in colorectal cancer. Indeed, some of the sub-modules were associated with chemotherapeutic sensitivity in cancer cells (Mod102, Mod107 and Mod109). Although we did not identified genes with specificity in CRC, these sub-modules may play a role in chemoresistance in CRC. For instance, we have validated the *PTGES* gene for its potential novel role in chemoresistance in CRC cells. Overall, the network-based approach successfully identified a robust predictive signature, which was tightly correlated with biological functions.

## Conclusions

Our study based on retrospective data identified a 11-PPI-Mod predictor, which showed a promising predictive value for survival benefit of adjuvant chemotherapy in stage II CRCs. The relatively small number of patients in the adjuvant chemotherapy group may limit the predictive efficiency. A prospective large-cohort study is suggested to validate the 11-PPI-Mod signature. Furthermore, the high-risk PPI modules/genes identified in this study might be novel therapeutic targets to increase chemo-sensitivity and improve outcome of patients.

## Additional files

Additional file 1: Tables S1-S4. indicate additional results of Cox regression analysis and genes involved in the 11-PPI-mod. **Figures S1-S4.** show additional information of data processing, feature selection and Kaplan-Meier analysis. (DOC 1262 kb)

## Abbreviations

11-PPI-Mod: 11 PPI sub-modules
AUC: Areas under the ROC curves
CI: Confidence interval
CRC: Colorectal cancer
dMMR: Defective DNA mismatch repair
FDR: False discovery rate
GEO: Gene expression omnibus
GO: Gene ontology
GSVA: Gene set variation analysis
GTOM: General topological overlap matrix
HPRD: Human protein reference database
LOH: Los of heterozygosity
MSI: Microsatellite instability
OOB: Out of bag
OS: Overall survival
pMMR: Proficient DNA mismatch repair
PPI: Protein-protein interaction
RFS: Relapse-free survival
ROC: Receiver operating characteristic
