# Supplementary material for: A network-based predictive gene-expression signature for adjuvant chemotherapy benefit in stage II colorectal cancer
Source: BMC Cancer. 2017 Dec 13;17:844. doi: 10.1186/s12885-017-3821-4 (PMC5729289; doi:10.1186/s12885-017-3821-4)
Supplement: Supplementary file 1 — indicate additional results of Cox regression analysis and genes involved in the 11-PPI-mod. Figures S1-S4. show additional information of data processing, feature selection and Kaplan-Meier analysis. (DOC 1262 kb) [file 12885_2017_3821_MOESM1_ESM.doc]

Table S1 Cox proportional hazards regression on RFS in the training dataset

| ^1^Variables |  | *n* (%) | Univariate Cox | |  | ^2^Interaction effect  with Chemotherapy | |
| --- | --- | --- | --- | --- | --- | --- | --- |
|  |  |  | HR(95%CI) | Pvalue |  | HR(95%CI) | Pvalue |
| Sex |  |  |  |  |  |  |  |
|  | Female | 85(40.1) | 1 |  |  | 1 |  |
|  | Male | 127(59.9) | 1.31(0.73-2.37) | 0.364 |  | 0.72(0.20-2.53) | 0.605 |
| Age |  |  |  |  |  |  |  |
|  | <70 ys | 109(51.4) | 1 |  |  | 1 |  |
|  | ≥70 ys | 103(48.6) | 1.11(0.63-1.94) | 0.723 |  | 0.86(0.23-3.26) | 0.822 |
| Location |  |  |  |  |  |  |  |
|  | distal | 147(69.3) | 1 |  |  | 1 |  |
|  | proximal | 65(30.7) | 0.84(0.45-1.59) | 0.591 |  | 0.26(0.03-2.19) | 0.213 |
| pT stage |  |  |  |  |  |  |  |
|  | T3 | 164(77.3) | 1 |  |  | 1 |  |
|  | T4 | 38(17.9) | 2.26(1.14-4.46) | 0.019 |  | 1.11(0.26-4.77) | 0.891 |
|  | N/A | 10(4.7) |  |  |  |  |  |
| Chemotherapy | |  |  |  |  |  |  |
|  | No | 162(76.4) | 1 |  |  | - | - |
|  | Yes | 50(23.6) | 1.4(0.76-2.57) | 0.276 |  | - | - |
| Mol.Type |  |  |  |  |  |  |  |
|  | C1 | 56(26.4) | 1 |  |  | 1 |  |
|  | C2 | 18(8.5) | 0.81(0.27-2.46) | 0.709 |  | 1.03(0.11-10.13) | 0.977 |
|  | C3 | 29(13.7) | 0.54(0.18-1.63) | 0.271 |  | 0(0-Inf) | 0.997 |
|  | C4 | 15(7.1) | 1.31(0.47-3.63) | 0.609 |  | 1.76(0.15-21.09) | 0.655 |
|  | C5 | 68(32.1) | 0.83(0.40-1.71) | 0.606 |  | 1.04(0.21-5.07) | 0.959 |
|  | C6 | 26(12.3) | 1.01(0.41-2.49) | 0.990 |  | 0.76(0.11-5.04) | 0.775 |
| Mut.BRAF |  |  |  |  |  |  |  |
|  | M | 5(2.4) | 1 |  |  | 1 |  |
|  | WT | 191(90.1) | 0.84(0.11-6.09) | 0.859 |  | - | - |
|  | N/A | 16(7.5) |  |  |  |  |  |
| Mut.KRAS |  |  |  |  |  |  |  |
|  | M | 72(34) | 1 |  |  | 1 |  |
|  | WT | 134(63.2) | 0.67(0.38-1.17) | 0.160 |  | 0.88(0.25-3.01) | 0.833 |
|  | N/A | 6(2.8) |  |  |  |  |  |
| Mut.TP53 |  |  |  |  |  |  |  |
|  | M | 65(30.7) | 1 |  |  | 1 |  |
|  | WT | 64(30.2) | 0.57(0.30-1.09) | 0.088 |  | 0.58(0.12-2.74) | 0.496 |
|  | N/A | 83(39.2) |  |  |  |  |  |
| Cluster |  |  |  |  |  |  |  |
|  | Cluster1 | 140(66) | 1 |  |  | 1 |  |
|  | Cluster2 | 72(34) | 1.75(0.99-3.06) | 0.052 |  | 7.52(1.75-32.28) | 0.007 |

^1^ ys, years; pT stage, pathological T stage; Mol.Type, molecular subtype from the original article; Mut.BRAF/Mut.KRAR/Mut.TP53, somatic mutation status of BRAF/KRAS/TP53; WT, wild type; M, mutation; N/A, not available. ^2^To test the interaction effect between each variable and Chemotherapy status.

Table S2 Cox proportional hazards regression on OS in the training dataset

| ^1^Variables |  | *n*(%) | Univariate Cox | |  | Interaction with Chemotherapy | |
| --- | --- | --- | --- | --- | --- | --- | --- |
|  |  |  | HR(95%CI) | Pvalue |  | HR(95%CI) | Pvalue |
| Sex |  |  |  |  |  |  |  |
|  | Female | 85(40.1) | 1 |  |  | 1 |  |
|  | Male | 127(59.9) | 1.06(0.63-1.78) | 0.832 |  | 0.81(0.24-2.73) | 0.731 |
| Age |  |  |  |  |  |  |  |
|  | <70 ys | 109(51.4) | 1 |  |  | 1 |  |
|  | ≥70 ys | 103(48.6) | 2.83(1.64-4.89) | 0.0002 |  | 0.27(0.07-1.08) | 0.064 |
| Location |  |  |  |  |  |  |  |
|  | distal | 147(69.3) | 1 |  |  | 1 |  |
|  | proximal | 65(30.7) | 0.73(0.4-1.32) | 0.298 |  | 0.42(0.05-3.54) | 0.424 |
| pT stage |  |  |  |  |  |  |  |
|  | T3 | 164(77.3) | 1 |  |  | 1 |  |
|  | T4 | 38(17.9) | 1.6(0.85-3.01) | 0.143 |  | 3.06(0.77-12.14) | 0.111 |
|  | N/A | 10(4.7) |  |  |  |  |  |
| Chemotherapy | |  |  |  |  |  |  |
|  | No | 162(76.4) | 1 |  |  | - | - |
|  | Yes | 50(23.6) | 0.87(0.47-1.58) | 0.637 |  | - | - |
| Mol.Type |  |  |  |  |  |  |  |
|  | C1 | 56(26.4) | 1 |  |  | 1 |  |
|  | C2 | 18(8.5) | 0.49(0.17-1.41) | 0.185 |  | 2.36(0.25-22.26) | 0.454 |
|  | C3 | 29(13.7) | 0.09(0.01-0.67) | 0.019 |  | 0(0-Inf) | 0.996 |
|  | C4 | 15(7.1) | 0.55(0.19-1.6) | 0.273 |  | 7.78(0.62-97.41) | 0.112 |
|  | C5 | 68(32.1) | 0.74(0.41-1.34) | 0.32 |  | 1.15(0.26-5.04) | 0.857 |
|  | C6 | 26(12.3) | 0.64(0.27-1.5) | 0.308 |  | 0.78(0.11-5.62) | 0.809 |
| Mut.BRAF |  |  |  |  |  |  |  |
|  | M | 5(2.4) | 1 |  |  | 1 |  |
|  | WT | 191(90.1) | 1.27(0.18-9.26) | 0.811 |  | - | - |
|  | N/A | 16(7.5) |  |  |  |  |  |
| Mut.KRAS |  |  |  |  |  |  |  |
|  | M | 72(34) | 1 |  |  | 1 |  |
|  | WT | 134(63.2) | 0.76(0.45-1.29) | 0.308 |  | 0.22(0.06-0.75) | 0.016 |
|  | N/A | 6(2.8) |  |  |  |  |  |
| Mut.TP53 |  |  |  |  |  |  |  |
|  | M | 65(30.7) | 1 |  |  | 1 |  |
|  | WT | 64(30.2) | 0.58(0.31-1.08) | 0.088 |  | 0.9(0.18-4.44) | 0.899 |
|  | N/A | 83(39.2) |  |  |  |  |  |
| Cluster |  |  |  |  |  |  |  |
|  | Cluster1 | 140(66) | 1 |  |  | 1 |  |
|  | Cluster2 | 72(34) | 0.88(0.52-1.51) | 0.651 |  | 6.54(1.7-25.15) | 0.006 |

^1^ ys, years; pT stage, pathological T stage; Mol.Type, molecular subtype from the original article; Mut.BRAF/Mut.KRAR/Mut.TP53, somatic mutation status of BRAF/KRAS/TP53; WT, wild type; M, mutation; N/A, not available. ^2^To test the interaction effect between each variable and Chemotherapy status.

Table S3 Cox proportional hazards regression on RFS in the validation dataset

| ^1^Variables |  | *n*(%) | Univariate Cox | |  | ^2^Interaction effect  with Chemotherapy | |
| --- | --- | --- | --- | --- | --- | --- | --- |
|  |  |  | HR(95%CI) | Pvalue |  | HR(95%CI) | Pvalue |
| Sex |  |  |  |  |  |  |  |
|  | Female | 40(47.1) | 1 |  |  | 1 |  |
|  | Male | 45(52.9) | 1.08(0.69-1.7) | 0.726 |  | 0.53(0.14-1.96) | 0.341 |
| Age |  |  |  |  |  |  |  |
|  | <70ys | 40(47.1) | 1 |  |  | 1 |  |
|  | ≥70ys | 45(52.9) | 0.85(0.54-1.34) | 0.479 |  | 0.91(0.24-3.37) | 0.884 |
| Location |  |  |  |  |  |  |  |
|  | distal | 41(48.2) | 1 |  |  | 1 |  |
|  | proximal | 44(51.8) | 1.45(0.92-2.30) | 0.111 |  | 0.52(0.13-2.13) | 0.36 |
| Chemotherapy | |  |  |  |  |  |  |
|  | No | 72(84.7) | 1 |  |  | - | - |
|  | Yes | 13(15.3) | 0.75(0.41-1.40) | 0.369 |  | - | - |
| Cluster |  |  |  |  |  |  |  |
|  | Cluster1 | 51(60) | 1 |  |  | 1 |  |
|  | Cluster2 | 34(40) | 0.77(0.48-1.23) | 0.275 |  | 8.42(2.15-33.03) | 0.002 |

^1^ ys, years; ^2^To test the interaction effect between each variable and Chemotherapy status.

Table S4. Genes involved in the 11 selected PPI sub-modules

| Gene  Symbol | Entrez  Gene ID | Module |  | Gene  Symbol | Entrez  Gene ID | Module |  | Gene  Symbol | Entrez  Gene ID | Module |
| --- | --- | --- | --- | --- | --- | --- | --- | --- | --- | --- |
| PCNA | 5111 | Mod102 |  | WNT2 | 7472 | Mod109 |  | NEXN | 91624 | Mod44 |
| POLQ | 10721 | Mod102 |  | RASA3 | 22821 | Mod303 |  | ACTR10 | 55860 | Mod44 |
| CCNO | 10309 | Mod102 |  | ELMO1 | 9844 | Mod303 |  | GAS7 | 8522 | Mod44 |
| POLD3 | 10714 | Mod102 |  | ASAP1 | 50807 | Mod303 |  | XIRP1 | 165904 | Mod44 |
| CHTF18 | 63922 | Mod102 |  | WIPF1 | 7456 | Mod303 |  | FGD4 | 121512 | Mod44 |
| UNG | 7374 | Mod102 |  | HCK | 3055 | Mod303 |  | PIAS4 | 51588 | Mod451 |
| POLD4 | 57804 | Mod102 |  | AGK | 55750 | Mod303 |  | KIF22 | 3835 | Mod451 |
| RFC4 | 5984 | Mod102 |  | IRF2BPL | 64207 | Mod303 |  | MRPL44 | 65080 | Mod451 |
| SEC23IP | 11196 | Mod102 |  | SPINK1 | 6690 | Mod371 |  | PLA2G15 | 23659 | Mod451 |
| DNMT1 | 1786 | Mod102 |  | TMPRSS15 | 5651 | Mod371 |  | HJURP | 55355 | Mod451 |
| HAND1 | 9421 | Mod107 |  | NRSN1 | 140767 | Mod371 |  | STMN4 | 81551 | Mod451 |
| HAND2 | 9464 | Mod107 |  | FBN1 | 2200 | Mod371 |  | MAPK8IP3 | 23162 | Mod481 |
| TAL2 | 6887 | Mod107 |  | LOXL1 | 4016 | Mod371 |  | DEFB4A | 1673 | Mod481 |
| TCF7L1 | 83439 | Mod107 |  | MFAP2 | 4237 | Mod371 |  | LY96 | 23643 | Mod481 |
| LMX1B | 4010 | Mod107 |  | ELN | 2006 | Mod371 |  | NDUFA2 | 4695 | Mod481 |
| PSMD9 | 5715 | Mod107 |  | PTGDS | 5730 | Mod431 |  | NOX4 | 50507 | Mod481 |
| TCF21 | 6943 | Mod107 |  | PTGER1 | 5731 | Mod431 |  | CNPY4 | 245812 | Mod481 |
| AEBP1 | 165 | Mod107 |  | PTGES | 9536 | Mod431 |  | CNN1 | 1264 | Mod522 |
| RALGAPA1 | 253959 | Mod107 |  | PTGER2 | 5732 | Mod431 |  | TRPV1 | 7442 | Mod522 |
| FERD3L | 222894 | Mod107 |  | PTGFR | 5737 | Mod431 |  | OBSCN | 84033 | Mod522 |
| SFRP2 | 6423 | Mod109 |  | PTGER3 | 5733 | Mod431 |  | MYO9B | 4650 | Mod522 |
| RYK | 6259 | Mod109 |  | SORBS1 | 10580 | Mod44 |  | ITPKA | 3706 | Mod522 |
| PORCN | 64840 | Mod109 |  | PARVA | 55742 | Mod44 |  | TRPV2 | 51393 | Mod522 |
| WNT7B | 7477 | Mod109 |  | SPTAN1 | 6709 | Mod44 |  | PRKRIP1 | 79706 | Mod665 |
| SFRP1 | 6422 | Mod109 |  | EPB41L2 | 2037 | Mod44 |  | CLUAP1 | 23059 | Mod665 |
| FZD6 | 8323 | Mod109 |  | SPTA1 | 6708 | Mod44 |  | ILF3 | 3609 | Mod665 |
| WNT4 | 54361 | Mod109 |  | GAS2 | 2620 | Mod44 |  | NOL4 | 8715 | Mod665 |
| WNT6 | 7475 | Mod109 |  | ADSS | 159 | Mod44 |  | CINP | 51550 | Mod665 |
| WNT1 | 7471 | Mod109 |  | CCIN | 881 | Mod44 |  |  |  |  |

**Figure S1**. A flowchart of gene-expression data processing in this study.

**Figure S2**. Feature selection for the classification of two sub-types of patients. (A) Predictive importance of the 44 candidate PPI sub-modules. The x-axis indicates the index of variables (PPI sub-modules), the y-axis indicates the importance of variables. The predictive importance of the true data (initial, black), 20 simulated data by shuffling samples (simulated, light blue), and mean of simulated data (red) are presented, respectively. (B) Predictive accuracy of different combination of variables. The x-axis represents number of variables used (different variable combinations), the y-axis represents the Out-of-Bag (OOB) error rate in the prediction of patients sub-types. The solid line indicates the true OOB error, and the dotted lines indicate the standard error of the OOB.

**Figure S3**. Kaplan-Meier analysis of OS stratified by Kras status and 11-PPI-mod predictor in the training dataset. Kaplan-Meier curves were compared between patients with (Adj.Ther.) or without (non-Adj.Ther.) adjuvant chemotherapy, in each sub-groups as indicated. The *p* value was estimated by log-rank test.


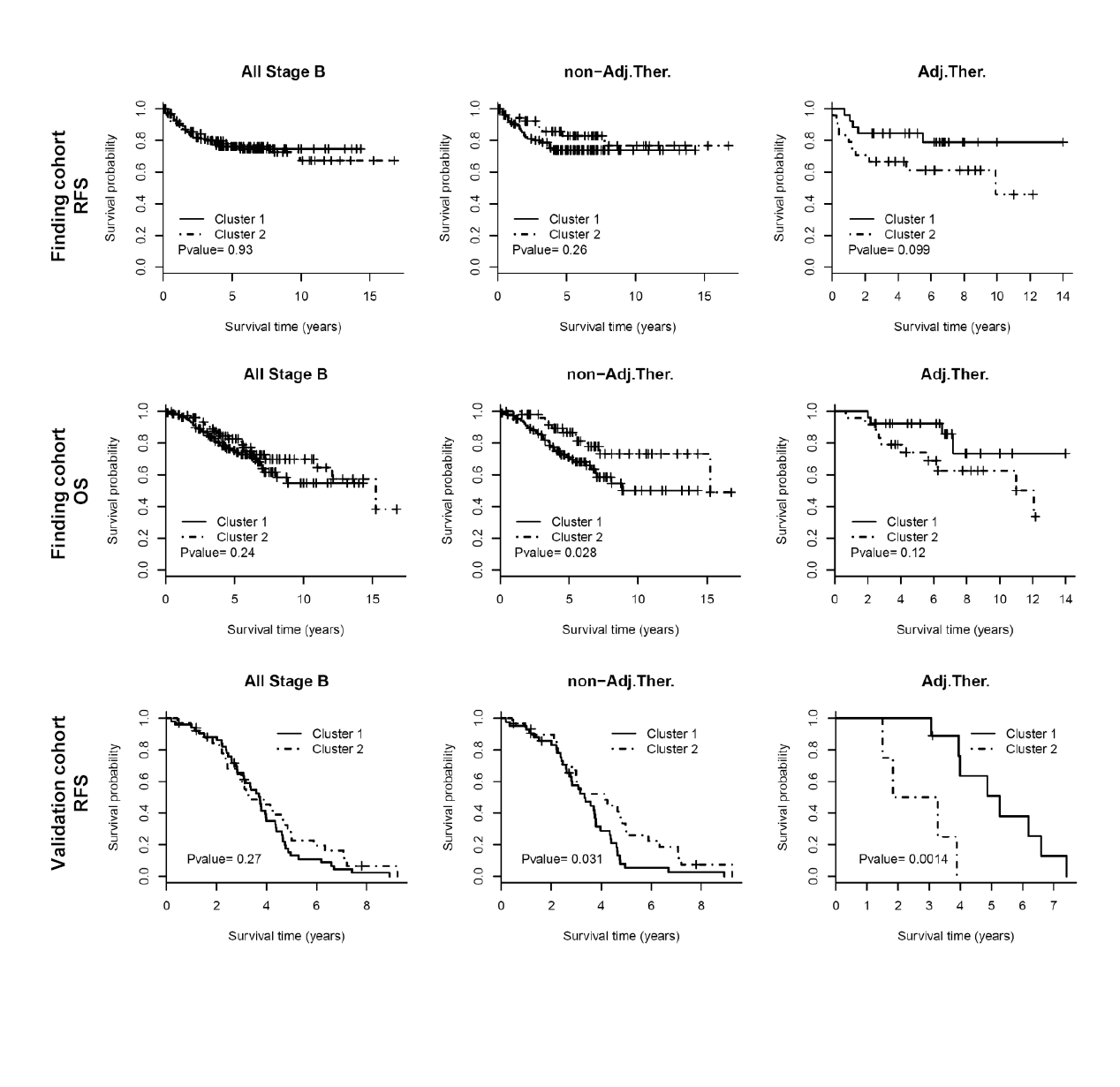


**Figure S4**. Prognostic analysis of 11-PPI-mod in the training dataset (GSE39582) and the validation dataset (GSE14333). Kaplan-Meier curves for RFS or OS were compared for the two clusters (Cluster 1 vs 2) classified by the 11-PPI-mod, stratified by chemotherapy treatment (non-Adj.Ther., without adjuvant therapy; Adj.Ther., with adjuvant therapy). The *p* value was estimated by log-rank test.
